# Fibroblastic variant of low-grade endometrial stromal sarcoma mimicking an inflammatory pseudotumor: a case report

**DOI:** 10.11604/pamj.2022.43.34.29749

**Published:** 2022-09-21

**Authors:** Anass Haloui, Nassira Karich, Achraf Miry, Amal Bennani

**Affiliations:** 1 Laboratory of Pathological Anatomy, Faculty of Medicine and Pharmacy of Oujda, Mohammed First University, Oujda, Morocco

**Keywords:** Low grade endometrial stromal sarcoma, fibroblastic variant, endometrial stromal sarcoma, case report

## Abstract

The World Health Organization currently divides endometrial stromal sarcomas into 4 different entities, based on their clinical and pathological features: endometrial stromal nodule, low-grade endometrial stromal sarcomas (LG-ESS), High-grade endometrial stromal sarcomas, and undifferentiated uterine sarcoma. The fibroblastic variant of LG-ESS is rare and is usually made of small tumoral cells of oval to fusiform shape, demonstrating low cytologic atypia and low mitotic activity, which can lead to confusion with a benign myofibroblastic proliferation. We hereby report a rare case of a fibroblastic variant of LG-ESS in a 37-year-old woman presenting abundant metrorrhagia, which was initially misdiagnosed as an inflammatory pseudotumor before proofreading in our laboratory, along with a review of a histological and immunohistochemical findings, aiming to help pathologists avoid this diagnosis pitfall.

## Introduction

Endometrial stromal sarcoma is a group of malignant tumors defined by a proliferation of tumoral cells resembling endometrial stromal cells [1,2]. They occur most commonly in the uterus, and occasionally in the ovary and peritoneum, often associated with endometriosis [3,4]. Low-grade endometrial stromal sarcoma (LG-ESS) is the most common form. Microscopic examination usually reveals a proliferation characterized by overt endometrial stromal differentiation and bland nuclear features, along with myometrial permeation. The fibroblastic variant of this tumor is rare and can be mistaken for a benign fibroblastic proliferation. Because of the rarity of this type of malignancy and given the lack of medical therapeutic progress, the only consistent approach to treatment is based on hysterectomy. Through this case, we report an example of this diagnosis pitfall, while highlighting the histological and immunohistochemical characteristics of these tumors.

## Patient and observation

**Patient information:** a 37-year-old woman, G0P0A0, non-smoker, non-alcoholic, with no medical or surgical history, who presented abundant metrorrhagia.

**Clinical findings:** physical examination found abdominal tenderness with uterine bleeding.

**Timeline of current episode:** November 2020: transvaginal sonography was performed revealing two masses located in the uterine isthmus. December 2020: an excision by hysteroscopy of one mass and a biopsy of the second were performed. Initial pathological examination was performed indicating an inflammatory pseudotumor. January 2020: tissue blocks were addressed to our laboratory for proofreading. We recommended a complete excision of the second mass. February 2020: the patient underwent a hysterectomy with bilateral salpingectomy (Table 1).

**Table 1:** timeline of current episode

November 2020	December 2020	January 2020	February 2020
Transvaginal sonography was performed revealing two masses located in the uterine isthmus.	An excision by hysteroscopy of one mass and a biopsy of the second were performed. Initial pathological examination was performed indicating an inflammatory pseudotumor	Tissue blocks were addressed to our laboratory for proofreading. We recommended a complete excision of the second mass.	The patient underwent a hysterectomy with bilateral salpingectomy.

**Figure 1:**
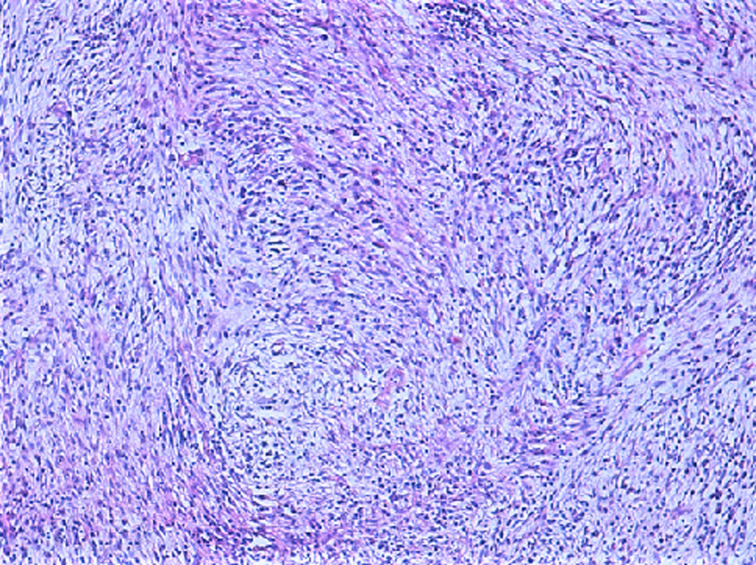
tumoral proliferation made of bland-looking spindle cells arranged in fascicles, provided with eosinophilic cytoplasm and ovoid nucleus with fine chromatin and discrete nucleoli; the stroma is inflammatory and focally myxoid

**Figure 2:**
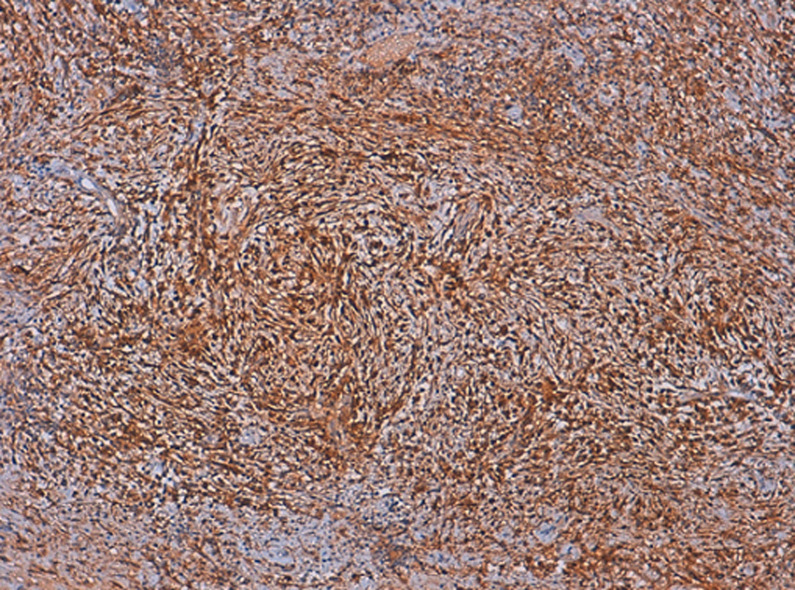
tumor cells expressing CD10

**Figure 3:**
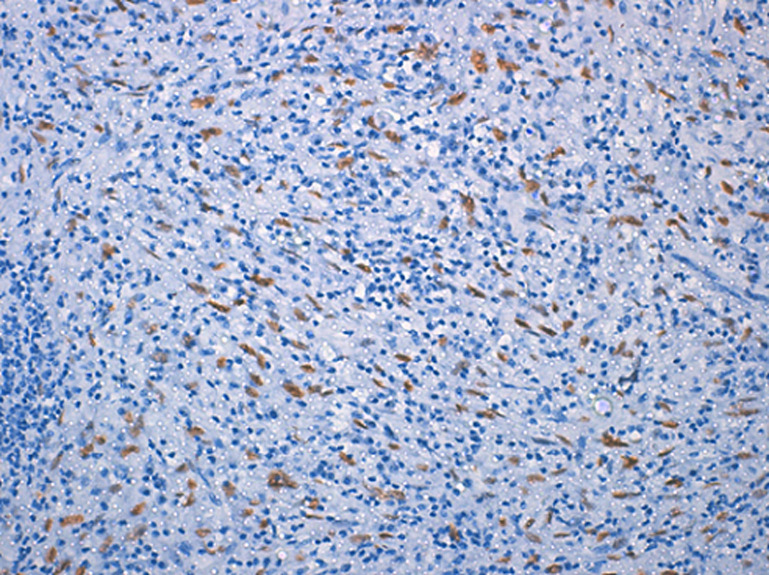
tumor cells showing nuclear expression of estrogen receptors

**Diagnostic assessment:** the histological examination of the tissue blocks revealed a tumoral proliferation made of bland-looking spindle cells arranged in fascicles, with eosinophilic cytoplasm and oval nucleus with fine chromatin and discrete nucleoli. They were displayed in an inflammatory and sometimes myxoid background (Figure 1). Mitotic count was low, estimated at 3 mitoses/10 fields at high power fields. Necrosis was absent. Tumor cells expressed CD10 (Figure 2) as well as hormonal receptors (Figure 3), without expression of Desmin or Anaplastic Lymphoma Kinase (ALK). Given the superficial nature of the samples, we could not assess the myometrial infiltration. Therefore, we recommended a complete removal of the second mass to determinate the degree of invasion. Thus, a hysterectomy with bilateral salpingectomy was performed. The gross examination found a whitish polypoid tumor located in the isthmus, of soft consistency, measuring 8x8.5x9cm, protruding into the uterine cavity with nodular myometrial permeation. Histological examination revealed the same proliferation described above, along with a permeative tongue-like pattern of myometrial invasion. Chest and abdominal CT-scans did not demonstrate metastasis.

**Diagnosis:** a diagnosis of fibroblastic variant of low grade endometrial stromal sarcoma was made, based upon histological and immunohistochemical findings

**Therapeutic interventions:** hysterectomy with bilateral salpingectomy.

**Follow-up and outcome of interventions:** to date, the patient remains asymptomatic and in excellent clinical condition, with no reported complications.

**Patient perspective:** “I feel very well”.

**Informed consent:** the patient gave informed consent.

## Discussion

Endometrial stromal tumors are considered among the rarest uterine neoplasms, representing 0.2% of all genital tract malignancies [5]. Low-grade endometrial stromal sarcoma is the most common, accounting for 80% of cases [6]. The fibroblastic variant is extremely rare, with only few cases reported in literature. They occur in perimenopausal women between 40 and 55 years old [7]. Some cases are associated with prolonged use of estrogen, tamoxifen, and pelvic irradiation [8]. The usual clinical features encountered are abnormal vaginal bleeding, or less commonly abdominal or pelvic pain. Metastatic tumor in an extra-uterine site is considered a rare presentation. These clinical features are consistent with the clinical symptoms of our patient, who presented abundant metrorrhagia.

In contrast to endometrial stromal nodules, endometrial stromal sarcomas tend to be poorly circumscribed on gross examination, forming intracavitary or intramural coalescent nodules invading the myometrium, frequently associated with worm-like plugs of tumor in the myometrial or parametrial veins [8]. They may also present as polypoid masses. The cut surface is typically fleshy, bulging and tan to yellow whitish. However, some cases may be deceptively well circumscribed, which should encourage thorough sampling of the tumor myometrial interface. Although the initial proofreading of paraffin-embedded tissue blocks in our case was highly suggestive of an endometrial stromal tumor, we had difficulty confirming the uterine tumor as LG-ESS, since no evidence of myometrial invasion nor vascular invasion was evident. Therefore, a hysterectomy was recommended. The macroscopic examination of the specimen revealed a whitish polypoid tumor located in the endometrium, protruding into the uterine cavity, with soft fleshy cut surface and nodular myometrial permeation.

On microscopic examination, LG-ESS commonly forms densely cellular islands of tumor cells, permeating the myometrium as irregular tongues, with or without lymphovascular invasion. The tumor cells typically display a uniform appearance, with scant cytoplasm, oval to fusiform nuclei and bland cytologic features, reminiscent of proliferative endometrial stromal cells. They often whorl around arteriole-like vessels forming a delicate arteriolar network in the stroma. Mitotic activity is usually low < 3/10 HPFs but may be brisk. Foci of foamy histiocytes, cystic change, necrosis and sometimes abundant amphophilic cytoplasm due to decidualization can be seen. Common variant features include smooth muscle differentiation, fibroblastic / myxoid changes and sex cord-like differentiation [9]. In the fibroblastic variant, tumor cells are arranged in a nodular, fascicular, or diffuse pattern possibly leading to a misdiagnosis of an inflammatory pseudotumor, as seen in our case. CD10 has good sensitivity for the diagnosis of LG-ESS, but the expression of this marker is not always diffuse and strong. A more specific marker is the Interferon-inducible transmembrane protein-1 (IFITM1). Other positive markers include hormone receptors, keratins, smooth muscle markers, and β-catenin [10].

The main differential diagnosis is endometrial stromal nodule. Since both conditions share the same histologic, immunohistochemical and molecular features, apart from myometrial and vascular invasion, a specific diagnosis cannot be made on a curettage specimen alone, as the margins of the tumor cannot be assessed. Therefore, extensive sampling of the tumor-myometrial interface is mandatory. Another differential diagnosis is highly cellular leiomyoma, which can also show irregular margin with the adjacent myometrium. However, it has a fascicular growth of spindled cells merging with the surrounding myometrium, rather than the typical tongue-like pattern of myoinvasion seen in endometrial stromal sarcomas. Furthermore, leiomyomas are positive for Desmine and H-Caldsemone. CD10/Desmine/H-caldsemon represent an optimal panel in dealing with a problematic case [10].

As reported above, the initial histological examination of the biopsy specimen before our proofreading, concluded to an inflammatory pseudotumor, which can display a fascicular pattern characterized by intersecting fascicles, or rarely a storiform pattern within an inflammatory background, mimicking a smooth muscle tumor, or less commonly an endometrial stromal tumor [10]. However, in the present case, we observed a proliferation arranged in fascicles with tumor cells showing more ovoid nuclei, rather than the typical elongated nuclei with wispy eosinophilic cytoplasm commonly found in inflammatory pseudotumor. Moreover, ALK was negative. The infiltrative pattern along with the immunopositivity for CD10 and hormone receptors, together with negativity for ALK and Desmin, ruled out the initial diagnosis of inflammatory pseudotumor and an eventual leiomyoma, pleading in favor of a fibroblastic variant of a low-grade stromal sarcoma. The current standard of therapy for endometrial stromal sarcoma is hysterectomy and bilateral salpingectomy. Chemotherapy has generally shown to be ineffective, whereas adjuvant radiotherapy has been successful in the management of such condition. Low-grade endometrial stromal sarcoma is generally a low-grade malignant neoplasm with an indolent clinical course and a good prognosis in the case of localized, hormone-sensitive lesions.

## Conclusion

The fibroblastic variant of low-grade endometrial sarcoma is a rare uterine neoplasm, which can be confused with an inflammatory pseudotumor by its morphological appearance.
